# Impacts of Sprouted Barley on Performance and Carcass Ultrasound Measurements in Angus-Influenced Steers During Backgrounding

**DOI:** 10.3390/ani16182889

**Published:** 2026-09-14

**Authors:** Zachary C. Crump, Anthony F. Alberto, Bailee L. Brown, Bryce W. Roholt, Lillian L. Okamoto, Ryan A. Spurling, Robert E. Ward, Brady M. Blackett, Robert L. Harding, Sanjeewa D. Ranathunga, Kara J. Thornton

**Affiliations:** 1 Department of Animal, Dairy and Veterinary Sciences, Utah State University, Logan, UT 84322, USA; zachary.crump@usu.edu (Z.C.C.); anthony.alberto@usu.edu (A.F.A.); bbean502@gmail.com (B.L.B.); bryce.roholt@usu.edu (B.W.R.); lillian.okamoto@usu.edu (L.L.O.); ryan.spurling@sdstate.edu (R.A.S.); 2 Department of Nutrition, Dietetics and Food Sciences, Utah State University, Logan, UT 84322, USA; robert.ward@usu.edu; 3 Renaissance Ag, Vineyard, UT 84057, USA; bradymblackett@gmail.com (B.M.B.); robhardingdvm@aol.com (R.L.H.); sanjeewadil@gmail.com (S.D.R.)

**Keywords:** backgrounding, beef cattle, feeding behavior, ruminal fermentation, vertical farming

## Abstract

The objective of this research was to determine the impacts of including sprouted barley (SB) in the diets of backgrounding beef steers. Angus-influenced steers (328 ± 6.59 kg) were assigned to one of four diets (n = 15/diet): a control (CON) diet, or a diet containing 5%, 10%, or 20% SB (DM basis)—replacing corn silage and some rolled barley. Individual intake and feeding behavior were monitored using Vytelle bunks. Body weight (BW) and ruminal fluid samples were collected on d 0, 30, and 60, while dry matter intake (DMI), feeding behavior, average daily gain (ADG), and gain-to-feed ratio (G:F) were monitored for 60 d. In addition, total amount of nutrients consumed (crude protein (CP), neutral detergent fiber (NDF), acid detergent fiber (ADF), net energy for maintenance (NEm), and net energy for gain (NEg)) were also assessed. Calf BW, ADG, and G:F were not impacted by diet (*p* ≤ 0.43). DMI and consumption of CP, NDF, ADF, NEm and NEg were all impacted by diet × day (*p* ≤ 0.01). The acetate:propionate, percentage of propionate and concentration of butyrate were each impacted by diet × day (*p* ≤ 0.007). Collectively, these findings suggest including SB in the diet modifies nutrient intake and ruminal fermentation in backgrounding steers.

## Simple Summary

Many inconsistencies in weather and land resource availability create a challenge for modern agricultural producers. Vertical farming systems can potentially provide a remedy for these issues by producing consistent, nutrient-dense feed with limited land and water needs. This research was designed to investigate the use of sprouted barley—grown in a vertical farming system—as an alternative feed for freshly weaned steers and its effects on performance. Cattle were fed different diets that consisted of a traditional ration for the region and three inclusion levels of sprouted barley, which replaced corn silage and a portion of rolled barley grain. Differences in dry matter intake, feeding behavior and ruminal activity between diets were assessed. However, these differences did not negatively impact overall production, implying that sprouted barley could be an effective alternative feedstuff for backgrounding steers when it is a cost-effective option.

## 1. Introduction

Global demand for agricultural products, including beef, is expected to rise substantially by 2050 as the global population continues to grow [1]. However, cattle producers face mounting challenges such as urban encroachment, diminishing arable land, and climate variability, each of which complicate consistent production of quality feeds. In the U.S over the past two decades, urbanization has contributed to a nearly 48-million-acre reduction in arable land [2]. Additionally, the main driver of decreased crop yields is drought, and the persistence of droughts is being seen with increased frequency, especially in the western U.S. [3,4]. These limitations highlight the need for alternative and sustainable feed systems that can support consistent production.

Vertical farming systems (VFSs) have emerged as an innovative technology, used for beef cattle, which could potentially mitigate the environmental and land use constraints associated with conventional feed production. These systems allow year-round cultivation in a controlled environment of cereal grains, such as barley, while using less water and land than conventional agricultural practices [5,6]. Sprouted barley (SB) is typically harvested six to seven days after seeding and is reported to contain approximately 12.6% dry matter (DM), 17.8% crude protein (CP), and 0.43 megacalorie (Mcal) net energy maintenance (NEm)/kg DM [7]. The nutritional composition and year-round availability make SB a potential supplement or replacement for traditional forages or grains at various stages of beef cattle production.

During beef production, the backgrounding phase is a critical transition period for freshly weaned cattle, bridging the gap between the cow–calf and feedlot phases. At this stage they are fed a primarily forage-based diet, with a small portion of cereal grains. During the backgrounding phase, forage-based diets support moderate growth while promoting ruminal development and the establishment of a stable ruminal microbiome [8]. Identifying alternative forages that can promote ruminal function and development while supporting animal performance is key to improving the overall efficiency and sustainability of the beef cattle industry.

While there is increasing interest in utilizing SB as a feedstuff, few studies have evaluated the impact of incorporating this feed into diets during the backgrounding stage of growing steers. Feed intake, growth, and nutrient utilization during backgrounding are key drivers of long-term production efficiency [8,9,10]. The objective of this study was to evaluate the effects of including varying levels of SB in the diets of backgrounding Angus-influenced steers on performance traits, ruminal health, and carcass characteristics, with the goal of determining the optimal inclusion rate to maximize production outcomes. It was hypothesized that by including SB at any level in the diets of backgrounding steers it would not negatively impact the performance, ruminal health, or carcass development of backgrounding steers.

## 2. Materials and Methods

### 2.1. Animal Care and Ethics

All animal care and use were approved for this study by the Institutional Animal Care and Use Committee (IACUC #13709). This study was conducted from October 2022 to January 2023 at the South farm at Utah State University located in Logan, UT, USA. Cattle were sourced from two locations, the Utah State University beef herd and a local producer in Nephi, Utah. All steers received identical vaccination protocols at weaning which included a 5-way, modified live vaccine (Bovi Shield Gold 5, Zoetis, Parsippany, NJ, USA), and an 8-way vaccine (One Shot Ulta 8, Zoetis, Parsippany, NJ, USA). Electronic (EID) tags were placed in the right ear of each animal. All steers were kept in four covered pens, each outfitted with two Vytelle feeding bunks (Vytelle, Kansas City, MO, USA). Sixty Angus-influenced steer calves (n = 60) were obtained for this study and initially stratified by weight (328 ± 6.59 kg) into one of four different pens. Each pen was then assigned one of four different diets, making this study a completely randomized design. During sampling days, steers were removed from each pen and walked to the handling facility located approximately 50 m away. Body weight (BW) was measured, and ruminal fluid was collected at approximately 9 a.m. each morning on d 0, 30, and 60.

### 2.2. Diet Formulation and Feeding Management

Diets consisted of a control diet (n = 15, CON)—which was representative of a typical backgrounding ration for the Intermountain West—or diets containing 5% (n = 15), 10% (n = 15), or 20% (n = 15) SB inclusion on a dry matter basis, which replaced a portion of corn silage and rolled barley grain (Table 1). Diets for this experiment were balanced to meet nutrient requirements of growing backgrounding steers. Care was taken to maintain isocaloric and isonitrogenous dietary formulations. However, nutrient analysis of the final diets revealed increased crude protein concentrations in the 10% and 20% SB diets relative to the formulated values. Sprouted barley was included at 5%, 10%, 20% of dietary dry matter (DM), based on unpublished preliminary research and consultation with industry professionals to reflect current production practices. Prior to mixing SB with other feedstuffs, SB was processed through a commercial woodchipper (Model # SC262-18VEMC, Parker Ford, PA, USA) to increase ration consistency. Ration components were mixed and delivered to Vytelle bunks with a mobile forage blender (Model # 480, Rissler TMR Mixers, Lancaster, PA, USA). Steers were fed rations ad libitum for the duration of the study (60 d) at approximately 6 a.m. each morning and 6 p.m. each evening. The nutrient content of the feed was analyzed using a wet chemistry protocol by Cumberland Valley Analytical (Waynesboro, PA, USA). Composite samples were collected every 30 d.

### 2.3. Growing Barley Sprouts

Utilizing a vertical farming system (Renaissance Ag, Vineyard, UT, USA), all barley sprouts were grown daily on-farm, as previously described [11]. In brief, the vertical farming unit maintained a temperature of 21 °C, with water application—via precision irrigation sprinklers—every hour for approximately 15 s to provide optimal growth conditions. Seeds were acquired from Western Seeds (Tremonton, UT, USA), in cooperation with the Intermountain Farmers Association (Salt Lake City, UT, USA). For optimal growth, a spring six-row barley seed was utilized for sprouting. Seeds were of the golden eye variety and were placed in the system without soaking or any additional pre-processing. Lights inside the system were used to stimulate the activation of chlorophyll. Seeds were sprouted for six d, then manually harvested and fed to cattle. At the end of each harvest cycle, each tray was washed, reseeded, and placed back in the vertical farming system for the next production cycle.

### 2.4. Animal Performance, Intake and Feeding Behavior

Individual feed intake and feeding behavior were recorded using Vytelle feed bunks as previously described [12,13]. In short, feeding behavior was characterized into two categorical traits, bunk visits (BVs), and feed bouts (FBs). A BV was defined as any occasion when an animal entered the feed bunk area, regardless of whether feed was consumed. An FB was recorded when an animal entered the feed bunk area and a minimum of 10 g of feed was consumed. The durations of each event (BV and FB) were also recorded and are reported as BV duration (BVDUR) and FB duration (FBDUR). Dry matter intake (DMI) was calculated at three different intervals: d 0–18, d 19–30, and d 31–60. Feed efficiency is presented as the gain-to-feed ratio (G:F), which was calculated by dividing the total weight gain by the total dry matter consumed (kg) over the 60 d study period. Nutrient intake for each animal was determined by multiplying individual DMI by the reported concentration of each nutrient in each steer’s respective diet.

### 2.5. Rumen Fluid Collection and Analysis

Ruminal fluid was collected on d 0, 30, and 60, at approximately 9 a.m., three hours after feeding. Collection and analysis were conducted as previously described [7]. In short, ruminal fluid was collected via esophageal tube. Fluid was filtered and pH was immediately assessed using a portable pH meter (Oakton pHTestr, Vernon Hills, IL, USA). A 1000 µL aliquot of filtered fluid was then transferred to a 1.5 mL microcentrifuge tube containing 200 µL of 25% metaphosphoric acid for stabilization before storage at −80 °C. At analysis, fluid was spiked with 0.85% crotonic acid as an internal standard. An eight-point calibration curve was established using a mixture containing seven volatile fatty acids. Acetic, propionic, and butyric acids ranged from 10 to 0.08 mM, whereas isobutyric, valeric, isovaleric, and caproic acids ranged from 5 to 0.04 mM. Calibration standards were then transferred to autosampler vials (Part number:5182-0716, Agilent, Santa Clara, CA, USA) and were analyzed using an Agilent 6890 gas chromatograph. The gas chromatograph was equipped with a ZB-FFAP column (30 m × 0.52 mm ID × 1.0 µm film thickness; Phenomenex, Torrance, CA, USA) and utilized a flame ionization detector. Samples were injected in a 50:1 split ratio, with the injector maintaining a temperature of 200 °C. Individual volatile fatty acids (VFAs) were identified by comparing retention times with internal standards. Regression equations were generated from calibration curves and were used to calculate VFA concentrations, which are expressed in millimolar (mM) concentrations.

### 2.6. Ultrasound Measurements

Ribeye area (REA) and 12th rib fat thickness (REFT) were evaluated on d 0, 30 and 60 via ultrasound (ExaGo, Universal Imaging, Bedford Hills, NY, USA). Canola oil was applied externally between the 12th and 13th rib as the acoustic couplant prior to the transducer being placed between the 12th and 13th rib to image both REA and REFT. All ultrasound images were analyzed using ImageJ software (version 1.54, National Institutes of Health and the Laboratory for Optical and Computational Instrumentation, University of Wisconsin, WI, USA) with REA being reported in cm^2^ and REFT in mm.

### 2.7. Statistical Analysis

Statistical analyses were performed using the PROC mixed procedure of SAS (SAS v 9.4, Cary, NC, USA). The model included diet, day, and the diet × day interaction as fixed effects, while cattle source and pen were included as random effects. Although diets were assigned at the pen level, individual feed intake was recorded using the Vytelle bunk system. Pen was not replicated within diet; therefore, diet effects were interpreted with this limitation in mind. A variance components covariance structure was selected, and residual diagnostics indicated that assumptions of normality and homogeneity of variance were adequately met under this model. Results are presented as least squares (LS) means ± the standard error of the mean (SEM). Steer BW, feeding behavior, ruminal pH, VFA profile, REA, REFT, average daily gain (ADG), and G:F were analyzed as repeated measures. When significant diet differences were detected (*p* < 0.05), the LS means were separated using Tukey–Kramer adjusted comparisons. The main effects of diet or day were not interpreted when a significant diet × day interaction was observed. Significance was declared at *p* ≤ 0.05, while tendencies were discussed between 0.05 < *p* ≤ 0.10.

## 3. Results

### 3.1. Animal Performance

**Figure 1:**
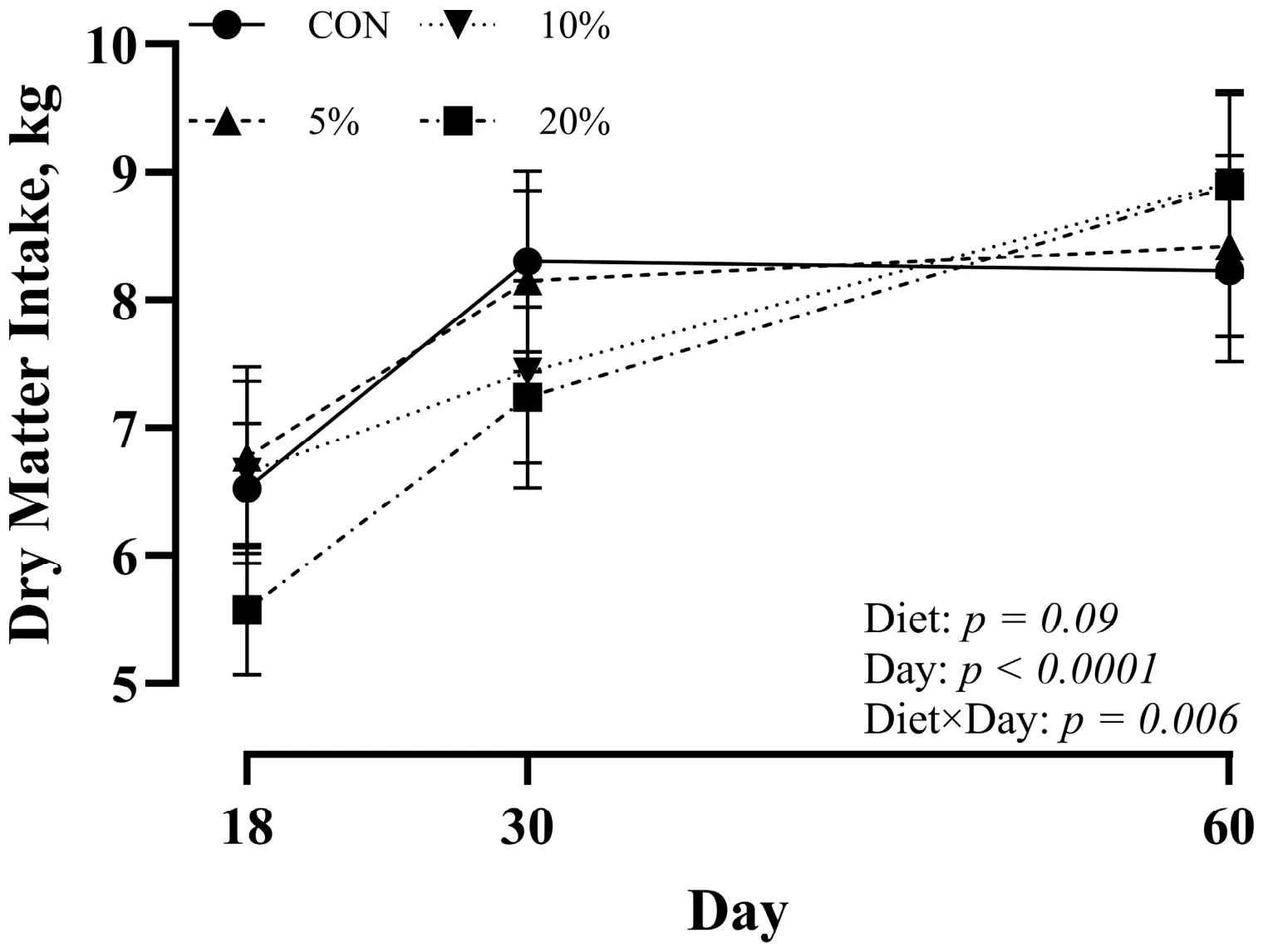
Steer performance data collected and calculated as described in the materials and methods. Sixty Angus steers were stratified based on initial body weight (BW; 328 ± 6.59 kg) into four different dietary groups (n = 15/group). A traditional ration for the region containing approximately 40% concentrate (DM) was used as the control (CON). Additionally, diets containing 5%, 10%, or 20% sprouted barley (SB; DM basis), replacing corn silage and a portion of the rolled barley, were fed in pens equipped with Vytelle bunks. Values presented at d 18 represent the average response from d 0–18, d 30 represents the average response from d 19–30, and d 60 represents the average response from d 31–60.

Over the course of this study, BW was not influenced by diet × day (*p* = 0.98) or diet (*p* = 0.18), but day (*p* < 0.0001) did impact BW. The BW of all animals, regardless of diet, increased (*p* < 0.0001) over time (Table 2). However, DMI was impacted by diet × day (*p* = 0.006), but no differences (*p* ≥ 0.26) between diets were present at any of the individual time points measured or across times (Figure 1). Diet did not impact either ADG or G:F (*p* ≥ 0.43; Table 2).

### 3.2. Feed Nutrient Intake

**Figure 2:**
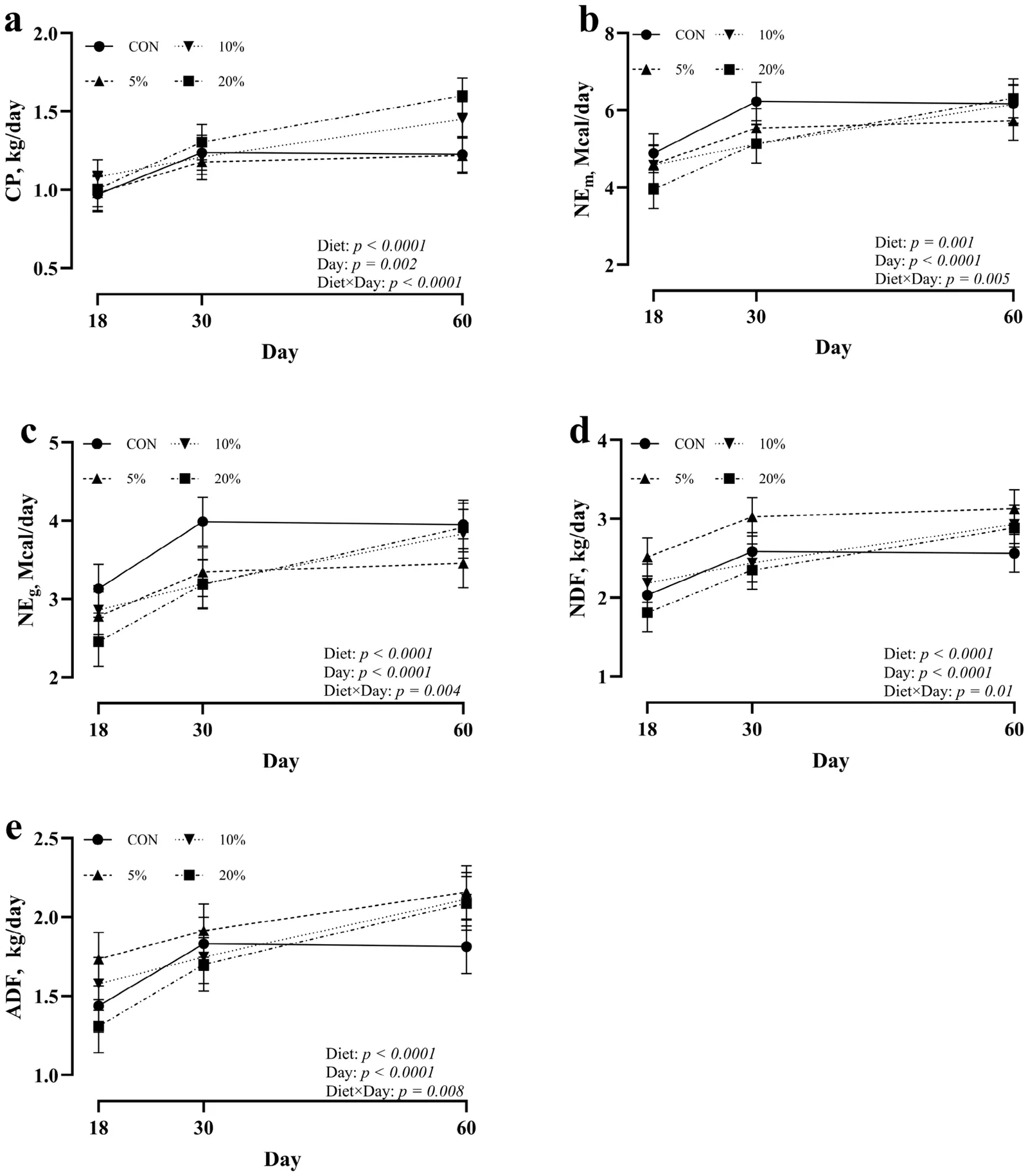
Steer nutrient intake data collected and calculated as described in the materials and methods. Sixty Angus steers were stratified based on initial body weight (BW; 328 ± 6.59 kg) into four different dietary groups (n = 15/group). A traditional ration for the region containing approximately 40% concentrate (DM) was used as the control (CON). Additionally, diets containing 5%, 10%, or 20% sprouted barley (SB; DM basis), replacing corn silage and a portion of the rolled barley, were fed in pens equipped with Vytelle bunks. Values presented at d 18 represent the average response from d 0–18, d 30 represents the average response from d 19–30, and d 60 represents the average response from d 31–60. The parameters assessed were crude protein (CP), kg/day (**a**), net energy maintenance (NEm), Mcal/day (**b**), net energy gain (NEg), Mcal/day (**c**), neutral detergent fiber (NDF), kg/day (**d**), acid detergent fiber (ADF), kg/day (**e**), using the MIXED procedure of SAS version 9.4.

The consumption of CP was impacted by diet × day (*p* = 0.0007); however, no differences (*p* ≥ 0.44) were seen between CON and any SB diets at any time point except on d 60 where 20% SB had increased (*p* < 0.0001) CP intake when compared to CON (Figure 2a). Daily NEm intake was affected by diet × day (*p* = 0.005) such that no differences (*p* ≥ 0.56) between NEm intake were evident between diets except on d 30 when 10% SB and 20% SB had decreased (*p* ≤ 0.009) intake (Figure 2b). The amount of NEg/day was altered by diet × day (*p* = 0.004), which can be described by 20% SB having decreased (*p* = 0.009) intake when compared to CON, as well as each SB diet having decreased (*p* ≥ 0.01) NEg intake on d 30 when compared to CON (Figure 2c). However, no other differences (*p* ≥ 0.19) in NEg intake were identified at any other time point when comparing CON to SB diets (Figure 2c). Neutral detergent fiber (NDF) was impacted by diet×day (*p* = 0.01), which can be described by 5% SB having increased (*p* ≤ 0.05) at each time point when compared to CON (Figure 2d). When comparing CON to each of the SB diets on d 18, 30, and 60, no differences (*p* ≥ 0.22) were observed (Figure 2d). Daily acid detergent fiber (ADF) intake had a diet×day (*p* = 0.008) interaction, such that on d 60, 5% SB had increased (*p* < 0.02) ADF intake when compared to CON (Figure 2e). On d 18, 5% SB tended to have increased (*p* = 0.08) ADF intake, and 10% SB showed a similar tendency to have increased (*p* = 0.07) intake compared with CON (Figure 2e).

### 3.3. Feeding Behavior

**Figure 3:**
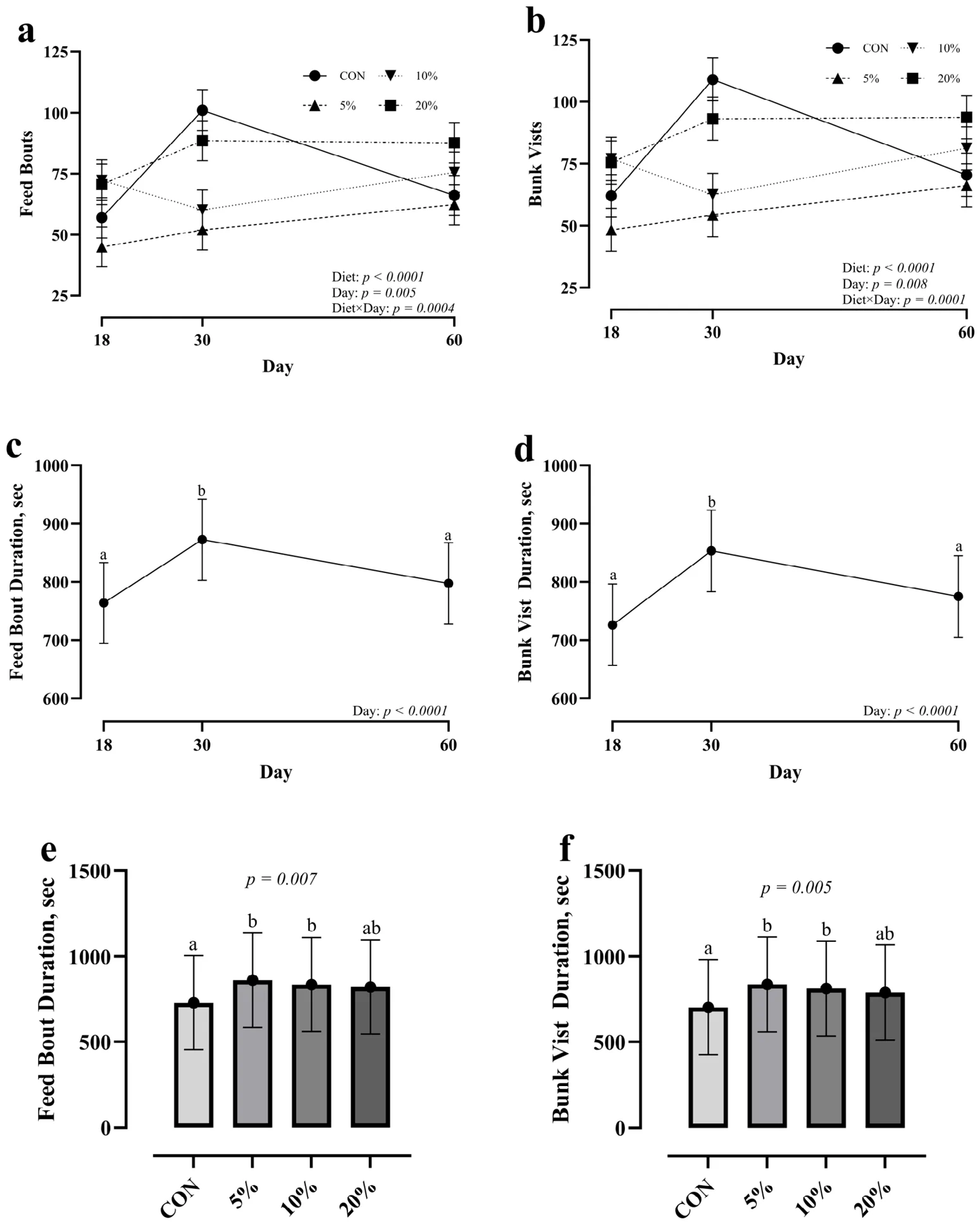
Steer feeding behavior data was collected as described in the materials and methods. Sixty Angus steers were stratified based on initial body weight (BW; 328 ± 6.59 kg) into four different dietary groups (n = 15/group). A traditional ration for the region containing approximately 40% concentrate (DM) was used as the control (CON). Additionally, diets containing 5%, 10%, or 20% sprouted barley (SB; DM basis), replacing corn silage and a portion of the rolled barley, were fed in pens equipped with Vytelle bunks. Values presented at d 18 represent the average response from d 0–18, d 30 represents the average response from d 19–30, and d 60 represents the average response from d 31–60. Feeding behavior was characterized into two categorical traits, bunk visits (BVs), and feed bouts (FBs). A BV was any occasion when an animal entered the feed bunk, regardless of whether feed was consumed. A FB was recorded when an animal entered the feed bunk and consumed a minimum of 10 g of feed. Data presented above includes steer FB (**a**), BV (**b**), FB duration over time (**c**), BV duration over time (**d**), FB duration by diet (**e**), and BV duration by diet (**f**). Data were analyzed using the MIXED procedure of SAS. When no significant diet × day effect was detected, only the significant main effect(s) of diet or day are presented. Data points with different letters (a, b, etc.) above them differ (*p* < 0.05) from one another within each panel of the figure.

For both FB and BV, a diet × day (*p* ≤ 0.0004) interaction was seen (Figure 3a,b). This was driven by decreases (*p* ≤ 0.009) in both FB and BV on d 30 for both 5% SB and 10% SB when contrasted to CON (Figure 3a,b). No other differences (*p* ≥ 0.41) were noted in FB and BV between diets at any time point when compared to CON. Neither FBDUR nor BVDUR were altered by diet × day (*p* ≥ 0.44); however, both diet (*p* ≤ 0.007), and day (*p* ≤ 0.005) altered both FBDUR (Figure 3c,e) and BVDUR (Figure 3d,f). Between d 18 and 30, an increase (*p* ≤ 0.006) in FBDUR and BVDUR was evident, but then BVDUR decreased (*p* = 0.05) from d 30 to 60, whereas FBDUR only tended to decrease (*p* = 0.07) in all animals during this period (Figure 3c,d). Diets of 5% SB and 10% SB had increased (*p* ≤ 0.04) FBDUR and BVDUR relative to CON, but 20% SB did not (*p* ≥ 0.10; Figure 3e,f).

### 3.4. Fermentation Dynamics

**Figure 4:**
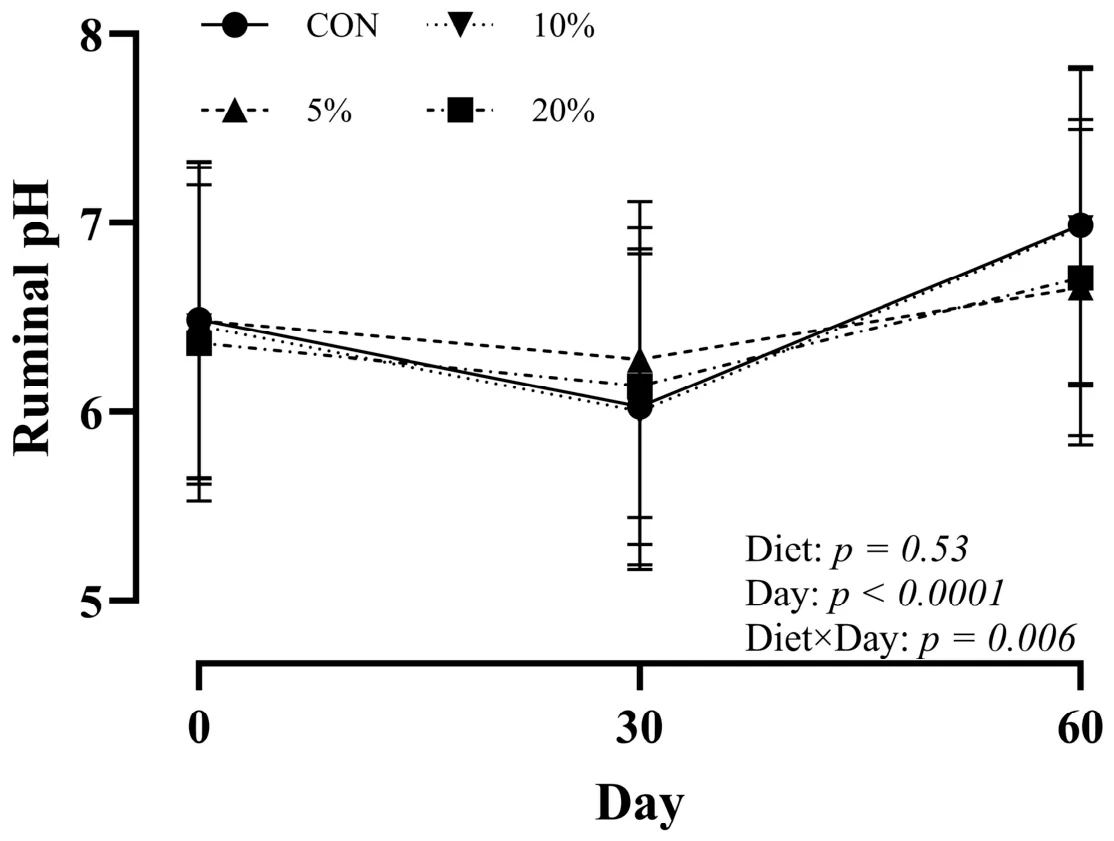
Steer ruminal pH data was collected as described in the materials and methods. Sixty Angus steers were stratified based on initial body weight (BW; 328 ± 6.59 kg) into four different dietary groups (n = 15/group). A traditional ration for the region containing approximately 40% concentrate (DM) was used as the control (CON). Additionally, diets containing 5%, 10%, or 20% sprouted barley (SB; DM basis), replacing corn silage and a portion of the rolled barley, were fed in pens equipped with Vytelle bunks. Data were analyzed using the MIXED procedure of SAS, with ruminal pH being analyzed as a repeated measure.

The ruminal pH was changed by diet × day (*p* = 0.006), but no differences (*p* ≥ 0.18) were seen between SB diets at any time relative to CON (Figure 4).

**Figure 5:**
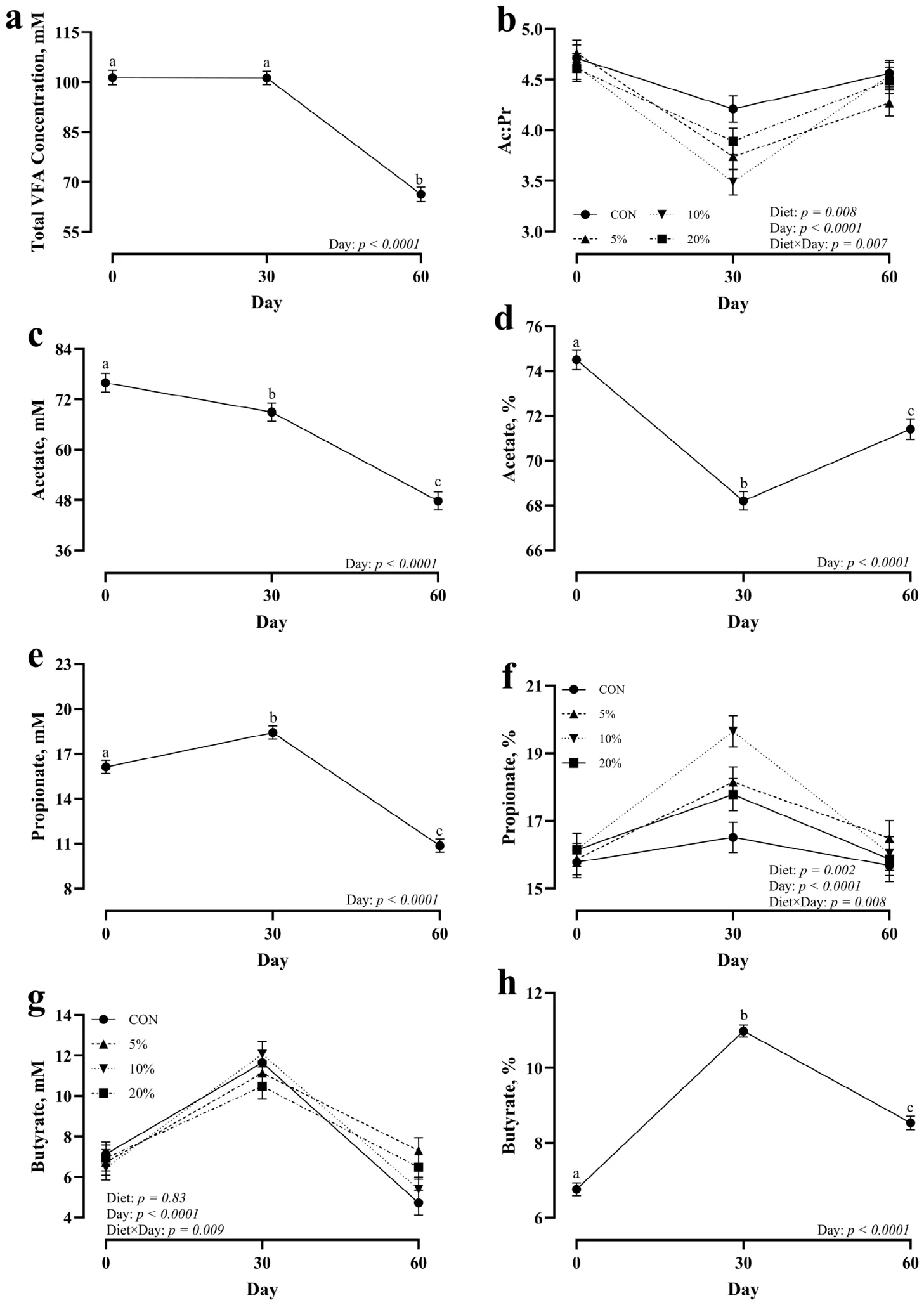
Sixty Angus steers were stratified based on initial body weight (BW; 328 ± 6.59 kg) into four different dietary groups (n = 15/group). A traditional ration for the region containing approximately 40% concentrate (DM) was used as the control (CON). Additionally, diets containing 5%, 10%, or 20% sprouted barley (SB; DM basis), replacing corn silage and a portion of the rolled barley, were fed in pens equipped with Vytelle bunks. All data were analyzed using the MIXED procedure of SAS version 9.4, with all volatile fatty acids (VFAs) being analyzed as a repeated measure. Measured parameters were total VFA (**a**), Ac:Pr (**b**), acetate concentration, mM (**c**), acetate percentage (**d**), propionate concentration, mM (**e**), propionate percentage (**f**), butyrate concentration, mM (**g**), and butyrate percentage (**h**). When no significant diet × day interaction was detected, only the significant main effect(s) of diet or day are presented. Data points with different letters (a, b, etc.) above them differ (*p* < 0.05) from one another within each panel of the figure.

Total concentration (mM) of VFA was not impacted by diet × day (*p* = 0.39) or diet (*p* = 0.98), but there was a change in concentration due to day (*p* < 0.0001; Figure 5a). There was no change (*p* = 0.99) in total VFA concentration (mM) from d 0 to 30, but then from d 30 to 60 there was a decrease (*p* < 0.0001; Figure 5a). The acetate-to-propionate ratio (Ac:Pr) was impacted by diet × day (*p* = 0.008), such that on d 30, 10% SB had a decreased (p = 0.002) Ac:Pr compared with CON, where 5% SB only tended (*p* = 0.07) to do the same (Figure 5b). No other differences (*p* ≥ 0.64) were observed between any SB diets and day (Figure 5b). The concentration (mM) of acetate had no diet × day (*p* = 0.10) or diet (*p* = 0.74) effects but was altered by day (*p* < 0.0001; Figure 5c). This can be seen by a decrease (*p* ≤ 0.007) in the concentration (mM) of acetate over the 60 d period (Figure 5c). Acetate percentage saw no diet × day (*p* = 0.18) interactions but a diet tendency (*p* = 0.07) was observed as well as a day (*p* < 0.0001) effect (Figure 5d). No differences (*p* ≥ 0.15) were seen when comparing SB diets to CON, but over time the percentage of acetate decreased for all diets (*p* < 0.0001; Figure 5d). Propionate concentration saw a diet × day (*p* = 0.06) and diet (*p* = 0.08) tendency, along with a day (*p* < 0.0001) effect (Figure 5e). There were no differences (*p* ≥ 0.12) seen when comparing SB diets to CON across time or when SB diets were contrasted to CON individually (Figure 5e). Propionate concentration increased (*p* = 0.001) the first 30 d then decreased (*p* < 0.0001) over the last 30 d for all diets (Figure 5e). The propionate percent that was found in the rumen was altered by diet × day (*p* = 0.002) such that on d 30, 10% SB had an increased (*p* < 0.0001) propionate percentage versus CON (Figure 5f). However, no other differences (*p* ≥ 0.10) were noted at any other time between SB diets and CON (Figure 5f). The concentration (mM) of butyrate was altered by diet × day (*p* = 0.009); however, no significance (*p* ≥ 0.15) was shown when comparing SB diets to CON across time (Figure 5g). Butyrate percent saw no diet×day (*p* = 0.80) or diet (*p* = 0.56) impact, but day (*p* < 0.0001) did which can be seen by an increase (*p* < 0.0001) from d 0 to 30, then a decrease (*p* < 0.0001) from d 30 to 60 in butyrate percentage (Figure 5h).

### 3.5. Ultrasound of REA and REFT

After ultrasound data was analyzed, it was found that both REA and REFT were not impacted by diet × day (*p* ≥ 0.85) or diet (*p* ≥ 0.26), but these values were impacted by day (*p* < 0.0001). This is described by both REA and REFT increasing (*p* < 0.0001) over time (Table 3).

## 4. Discussion

Beef producers continue to face challenges associated with feed availability, rising input costs, and increasing environmental variability, highlighting the need for alternative feed resources that can support animal performance while improving production sustainability [1,14]. Vertical farming systems offer a potential solution by producing SB, a nutrient-dense feedstuff that can be grown year-round with reduced reliance on traditional land and water inputs [11].

### 4.1. Animal Performance

As a main economic driver, animal performance is what is used to determine the viability of changes in production practices [15,16]. Therefore, ADG and G:F are influenced by numerous factors, including DMI, genetics, management practices, animal health, weather conditions, and diet composition [17,18,19]. In the current study, there were no performance differences in G:F, BW, REA, or REFT, even though SB tended to impact DMI by decreasing intake in the beginning of the study. In other feedlot research, a significant decrease in DMI was observed in finishing cattle that was large enough to affect G:F; however, no changes were observed in other performance metrics [20]. Producers continue to look for flexibility in feeding options and SB represents a viable alternative feed source for growing steers. The lack of differences in BW, ADG, or feed efficiency is particularly important, as it indicates that SB can be incorporated into cattle diets without compromising performance. This alternative option is critical in today’s production environment, where feed prices remain highly volatile [21] and alternative feed resources that stabilize feed costs and supply can offer a competitive advantage [22] depending on the economic viability of VFSs. Although no statistical differences in DMI were detected between diets at individual time points, cattle receiving the 20% SB diet showed a numerically greater increase in intake over the course of the study. One possible explanation is the lower dry matter concentration of the SB diets—particularly the 20% inclusion, which contained approximately 21% less DM than the control diet. Cattle generally regulate feed intake to satisfy dry matter and energy requirements rather than as-fed intake [23]. Therefore, the greater moisture content of SB may have encouraged animals to consume more feed on an as-fed basis while maintaining similar dry matter and energy intake. Similar responses have been reported in dairy cattle fed diets with increased moisture content, although published results remain inconsistent [24,25,26]. Reducing diet DM from 81% to 64% did not impact DMI but did increase NDF intake and milk fat percent [25]. Other impacts of adding water to the ration included reduced ration sorting [25,27], which increased DMI [27], but other research noted decreased DMI when ration DM was 51% or below during high ambient air temperatures [26]. These findings are inconsistent and unclear on the direct impacts of dietary DM composition on DMI in dairy cattle. Additionally, moisture added by sprouted grains is nutritionally different than regular water, which could impact the nutritional composition of the diet and performance. These findings suggest that the metabolic changes induced by sprouting were not substantial enough to alter nutrient supply to the animal. Although nutrient intake differed at several points, cattle appeared to maintain growth, indicating that SB could replace a portion of conventional feed without compromising production during the backgrounding phase. Other than work done by this research group [20], no other research to date has evaluated the impacts of dietary DM on feedlot cattle.

### 4.2. Feeding Behavior

Previous research has shown that increased BV and FB are associated with greater DMI and improved growth rates. [12]. Other research notes that animals with increased ADG spend approximately 8% more time at the bunk when compared to decreased ADG animals [28,29]. Based on this literature, it would suggest that all SB cattle would see an increase in performance as they spent more time at the bunk. However, no differences in any performance metric were seen (BW, G:F, ADG, DMI). The greater time spent at the bunk by cattle receiving SB diets was likely a behavioral response to the lower dietary DM concentration discussed previously. Animals may have required additional time to consume equivalent amounts of DM, resulting in increased bunk visits and feed bouts without improving overall growth performance. While these changes in feeding behavior did not negatively affect performance, the lower DM composition of SB diets could still influence how long animals spend at the bunk, possibly through effects on rumen fill, rumination, or feeding rate. Aside from studies conducted in dairy cattle and previous work from our research group [20,25,26,27] no other known research has investigated the impact of dietary DM composition on feeding behavior.

### 4.3. Feed Nutrient Intake

In previous studies in growing wethers and cow–calf pairs it was found that by adding SB to a ration, an increase in dietary CP could be achieved [7,11]. In this study, however, the opposite was found to be true which aligns with other SB feedlot research [20]. The increased CP intake observed in the 10% and 20% SB diets was primarily driven by their increased CP concentrations in the diets, due to increased SB in the diet, rather than differences in animal behavior. Additionally, DMI for the 10% SB and 20% SB diets increased over time relative to the control, contrary to reports in dairy cattle where increased dietary moisture reduced DMI and increased ration sorting behavior [24]. This discrepancy may bay be attributed to the differences between lactating dairy cows and growing backgrounding steers, whose nutrient requirements and feeding behavior differ considerably. Additionally, a study in growing wethers found that there was a similar decrease in DMI and subsequent energy consumption as the percentage of SB increased in the diet [11]. It was noted that this again is likely due to an overall decrease in DMI because of the increased moisture content of SB [7,11]. In the current study, the same conclusion can be drawn for each nutrient excluding CP. Each diet containing SB increased CP intake, with the greatest CP consumption being observed at a 20% SB inclusion rate. It is postulated that at such a small inclusion of SB, the DM percent of the ration did not cause a decrease in DMI to negatively affect energy consumption while altering nutrient intake and ruminal fermentation.

### 4.4. Ruminal Fermentation Dynamics

The physical and biochemical changes that occur during grain sprouting have the potential to alter ruminal fermentation. During germination, barley utilizes stored starch reserves to support seedling growth, resulting in starch reductions of up to 18.5% compared with the unsprouted grain [30,31,32,33]. This reduction is primarily driven by the activation of endogenous amylase enzymes, which hydrolyze starch into readily available soluble sugars that fuel sprout development [30,34]. As the shoot and root tissues develop, nutrient composition also changes. Starch has been reported to be 187% greater in the shoot fraction than in the root fraction, and the sprouted biomass contains greater concentrations of structural carbohydrates—including cellulose—than the original grain [30]. Collectively, these biochemical and compositional changes may alter the substrates available to ruminal microorganisms, potentially contributing to the differences in ruminal fermentation observed in the present study.

Testing ruminal pH along with VFA composition helps in understanding the impacts these changes in diet bioavailability can have on fermentation and ruminal health. Differences were noted in some VFA compositions; however, all differences that were noted were still within typical, acceptable ranges [35,36], which aligns with an acceptable ruminal pH across the diets [37]. One notable difference is an increase in the propionate percentage of animals that received SB, which was reflected by the decreased Ac:Pr ratio at d 30. As propionate is the primary driver of gluconeogenesis in cattle [38,39], increased propionate production may be of particular interest to feeders in finishing systems. Greater glucose availability from propionate can enhance intramuscular fat deposition, a key economic driver in beef production [40,41]. Glucose serves as a critical substrate for fatty acid synthesis and intramuscular fat accretion [40]. Diets that promote propionate production have the potential to influence carcass quality and overall value [40]. However, a feedlot study saw an increase in 12th rib fat thickness when a 20% SB inclusion diet was fed, but it was not correlated with an increase in marbling [20]. Additionally, an increase in backfat is associated with increased yield grades which can have negative economic repercussions [42,43].

### 4.5. Ultrasound of REA and REFT

Despite changes in nutrient intake and ruminal fermentation, muscle and fat deposition remained similar among diets. These changes in nutrient intake such as protein and energy consumption can have impacts on ADG which could result in decreased muscle growth and fat deposition [44]. No differences were observed in REA or REFT, suggesting that the relatively low inclusion rates of SB and the 60 d backgrounding period were insufficient to alter nutrient partitioning toward muscle or adipose tissue deposition. These findings agree with previous work reporting no differences in carcass characteristics when cattle were fed diets containing 20% SB (DM basis) [20]. One possible explanation is that biochemical changes that occurring during sprouting increase nutrient availability, allowing cattle to maintain growth despite differences in nutrient intake, although digestibility was not directly measured in the present study [45,46]. From an applied standpoint, these findings suggest that incorporating SB into the diets of backgrounding steers is unlikely to impact muscle growth or adipose deposition at an inclusion rate of up to 20% dietary DM.

## 5. Conclusions

Although producers are continuing to face challenges such as drought and urbanization, the use of VFSs may be an alternative option to maintain consistent quality feed for cattle. The inclusion of SB in the diet did not negatively affect production performance in backgrounding Angus-influenced steers. While differences in nutrient intake, ruminal pH, and VFA profiles were observed, these changes did not translate into reductions in BW gain, G:F, or carcass traits. These findings indicate that SB can be incorporated into the diets of backgrounding steers as an alternative feed source without compromising animal performance. Further studies with an increased sample size are needed to validate this study. Additionally, the economic viability of VFSs needs to be assessed at this stage in production to determine the feasibility of these systems.

## Figures and Tables

**Table 1:** Diet composition and nutrient analysis of diets fed to growing steers.

Diet Composition	CON ^1^, %	5% SB ^2^, %	10% SB ^3^, %	20% SB ^4^, %
Alfalfa	50.00	52.90	57.10	47.90
Rolled Barley	30.00	31.60	30.90	27.50
Sprouted Barley	0.00	5.30	10.00	22.00
Corn Silage	18.30	8.50	0.00	0.00
Mineral	1.50	1.80	1.90	2.60
**Nutrient Composition, % DM**				
DM ^5^	64.60	52.55	54.30	43.75
CP ^6^	14.90	14.45	16.25	17.95
NE_m_^7^, Mcal/kg	0.75	0.68	0.69	0.71
NE_g_^8^, Mcal/kg	0.48	0.41	0.43	0.44
NDF ^9^	31.15	37.20	32.85	32.50
ADF ^10^	22.05	25.65	23.70	23.50
Ash	7.79	8.55	9.35	9.40

^1^ CON, diet consisted of a typical ration for the Intermountain West, a 30% rolled barley concentrate; ^2^ 5% sprouted barley (SB), diet consisted of the same diet as control but corn silage and some rolled barley was replaced with SB at 5% dry matter (DM) inclusion; ^3^ 10% SB, diet consisted of the same diet as control but corn silage and some rolled barley was replaced with SB at 10% DM inclusion; ^4^ 20% SB, diet consisted of the same diet as control but corn silage and some rolled barley was replaced with SB at 20% DM inclusion; ^5^ DM, dry matter; ^6^ CP, crude protein; ^7^ Net energy maintenance (NE_m_), megacalorie (Mcal) per kilogram of feed; ^8^ Net energy gain (NE_g_), Mcal per kilogram of feed; ^9^ NDF, neutral detergent fiber; ^10^ ADF, acid detergent fiber.

**Table 2:** Steer performance and ultrasound measurements.

	Dietary Treatments ^1^						*p*-Value	
Measurement	CON ^1^	5% SB ^2^	10% SB ^3^	20% SB ^4^	SEM	Diet	Day	Diet × Day
BW, kg ^5^	370.97	348.63	349.40	366.04	12.77	*0.18*	*<0.0001*	*0.98*
ADG, kg ^6^	2.14	2.28	2.10	2.27	0.43	*0.84*	*-*	*-*
G:F ^7^	0.13	0.13	0.12	0.15	0.01	*0.43*	*-*	*-*

^1^ CON, diet consisted of a typical ration for the Intermountain West, a 30% rolled barley concentrate; ^2^ 5% sprouted barley (SB), diet consisted of the same diet as control but corn silage and some rolled barley was replaced with SB at 5% dry matter (DM) inclusion; ^3^ 10% SB, diet consisted of the same diet as control but corn silage and some rolled barley was replaced with SB at 10% DM inclusion; ^4^ 20% SB, diet consisted of the same diet as control but corn silage and some rolled barley was replaced with SB at 20% DM inclusion; ^5^ BW, body weight, ^6^ ADG, average daily gain; ^7^ G:F, gain to feed efficiency.

**Table 3:** Steer ultrasound measurements.

	Dietary Treatments ^1^						*p*-Value	
Measurement	CON	5% SB	10% SB	20% SB	SEM	Diet	Day	Diet × Day
REA ^2^, cm^2^	35.50	34.71	33.64	35.61	1.12	*0.26*	*<0.0001*	*0.85*
REFT ^3^, mm	1.39	1.37	1.48	1.37	0.25	*0.63*	*<0.0001*	*0.95*

^1^ CON, diet consisted of a typical ration for the Intermountain West, a 30% rolled barley concentrate; 5% sprouted barley (SB), diet consisted of the same diet as control but corn silage and some rolled barley was replaced with SB at 5% dry matter (DM) inclusion; 10% SB, diet consisted of the same diet as control but corn silage and some rolled barley was replaced with SB at 10% DM inclusion; 20% SB, diet consisted of the same diet as control but corn silage and some rolled barley was replaced with SB at 20% DM inclusion; ^2^ REA, ribeye area; ^3^ REFT, 12th rib fat thickness.

## Data Availability

The data presented in this study are available from the corresponding author upon reasonable request.

## Abbreviations

The following abbreviations are used in this manuscript:

ADG	Average daily gain
ADF	Acid detergent fiber
BW	Body weight
BV	Bunk Visit
BVDUR	Bunk Visit Duration
CON	Control diet
EID	Electronic identification tag
FB	Feed Bout
FBDUR	Feed Bout Duration
G:F	Gain to feed ratio
NDF	Neutral detergent fiber
REA	Ribeye area
REFT	12th rib fat thickness
SB	Sprouted barley
TMR	Total mixed ration
VFA	Volatile fatty acid
